# Proteomic profiling of a patient with cutaneous melanoma metastasis regression following topical contact sensitizer diphencyprone and immune checkpoint inhibitor treatment

**DOI:** 10.1038/s41598-022-27020-1

**Published:** 2022-12-26

**Authors:** Joseph Han, Aneesh Agarwal, Jade N. Young, Shayan Owji, Yen Luu, Dina Poplausky, Daniel Yassky, Yeriel Estrada, Jonathan Ungar, James G. Krueger, Nicholas Gulati

**Affiliations:** 1 Department of Dermatology, Icahn School of Medicine at Mount Sinai, 5 East 98th Street, New York, NY 10029 USA; 2 School of Medicine, University of Missouri-Kansas City, Kansas City, MO USA; 3 Laboratory for Investigative Dermatology, The Rockefeller University, New York, NY USA

**Keywords:** Melanoma, Proteomic analysis

## Abstract

Immune checkpoint inhibitors (ICIs) such as pembrolizumab have revolutionized the treatment of advanced melanoma, but many patients do not respond to ICIs alone, and thus there is need for additional treatment options. Topical immunomodulators such as diphencyprone (DPCP) also have clinical use in advanced melanoma, particularly in the treatment of cutaneous metastases. In a previous report, we characterized the enhanced clinical response to dual agent immunotherapy with pembrolizumab and DPCP in a patient with cutaneous melanoma metastases. To improve mechanistic understanding of this response, we analyzed proteomic data using the Olink immuno-oncology panel of 96 biomarkers from tissue and serum samples of this patient throughout his treatment course. Particular attention was paid to programmed death-1 (PD-1), programmed death-ligand 1 (PD-L1), and lymphocyte-activation gene 3 (LAG-3) given they are all targeted by ICIs in clinical practice. These proteins were upregulated during the period of DPCP monotherapy, then downregulated during pembrolizumab monotherapy, and then robustly upregulated again during dual therapy. Although not exclusively, the induction of checkpoint inhibitor proteins in the presence of DPCP suggests potential synergy between this agent and ICIs in the treatment of cutaneous melanoma metastases. Large-scale investigation is warranted to further evaluate this potential novel combination therapeutic approach.

## Introduction

Immune checkpoint inhibitors (ICIs) represent a relatively new class of drugs that function to enhance antitumor immunity^1^. Clinical use in melanoma treatment has shown particular promise, and ICIs are now standard of care for patients with advanced melanoma. In those with cutaneous metastases, complete resolution with ICIs is demonstrated in up to 20%, with a less than 10% 5-year risk of relapse^2^. Although their overall success has revolutionized the treatment of advanced melanoma, the majority of patients do not respond to ICI treatment alone^2^.

Topical immunotherapy in the form of contact sensitizers, such as diphencyprone (DPCP), has demonstrated at least partial cutaneous melanoma metastasis regression in up to 84% of patients across multiple studies^3,4^. In some cases, further disease progression still occurs despite treatment with contact sensitizer monotherapy. A prior two-patient case series has shown that these treatment failures may be mitigated by dual therapy induction with nivolumab^5^, a programmed death-1 (PD-1) inhibitor approved for many solid tumors^6^. These responses indicate that dual therapy with multiple immunogenic agents may have superior efficacy than any one immunotherapy alone. A more recently approved PD-1 inhibitor, pembrolizumab, has demonstrated successful outcomes similar to those of nivolumab monotherapy^7^, but has not been examined in detail as part of a dual therapeutic regimen with contact sensitizers.

**Figure 1:**
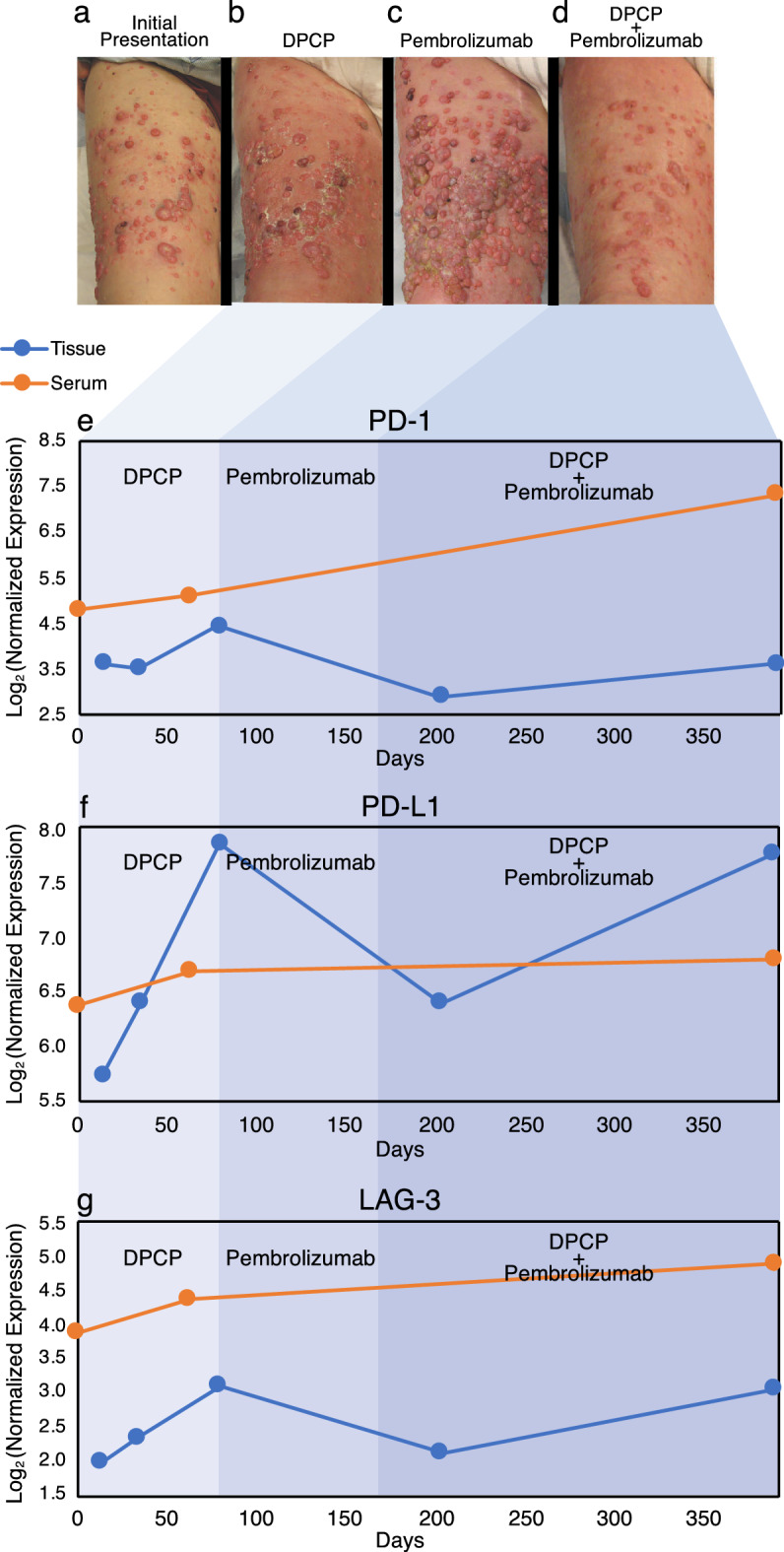
Responses of cutaneous melanoma metastases to topical DPCP, pembrolizumab, and a combination of both treatments. A photograph of the patient’s right anterior thigh before any treatment shows numerous amelanotic cutaneous melanoma metastases (**a**). Topical DPCP monotherapy (**b**) led to a mixed tumor response. During pembrolizumab monotherapy (**c**) there was rapid progression of disease. During dual therapy with pembrolizumab and DPCP, dramatic regression of cutaneous melanoma metastases was observed. Panels **a**–**d** adapted from Gulati et al.^4^ © 2016 John Wiley & Sons A/S. Published by John Wiley & Sons Ltd. Line plots of protein levels in serum and tissue of immune checkpoint markers PD-1 (**e**), PD-L1 (**f**), and LAG-3 (**g**) are shown correlating with the various phases of the patient’s treatment regimen.

We previously reported on the successful treatment of cutaneous melanoma metastases using a dual therapeutic regimen of pembrolizumab and DPCP^4^, despite previous lack of response to either agent alone (Fig. 1, a-d). In this previous report, clinical improvement was hypothesized to be attributable to synergistic drug activity. DPCP induces PD-1 and programmed death-ligand 1 (PD-L1) gene expression^8^, and pembrolizumab has reported mechanistic dependence on these checkpoint inhibitor proteins^4^. Given the successful clinical response, we sought to provide further evidence supporting this hypothesis through analysis of tissue and serum proteomic data from the same patient.

The study participant was a 93 year old male with metastatic melanoma and diffuse involvement of the right lower extremity. The patient did not have any metastases at distant sites or any non-cutaneous disease. Biopsy samples were taken at baseline, Day 80 (DPCP monotherapy), Day 202 (pembrolizumab monotherapy), and Day 385 (dual therapy). DPCP was discontinued after the final time point due to the patient transitioning to comfort care measures only, and the subject passed away shortly after for reasons thought not to be related to his cancer or cancer therapies.

## Results

### Variations in melanoma metastasis clinical responses during the dual therapy treatment course

At baseline, the patient presented with extensive in-transit metastases of the right lower extremity (Fig. 1a). Following DPCP topical monotherapy, a diffuse delayed-type hypersensitivity reaction occurred with mixed tumor regression response (Fig. 1b). Although some lesions appear larger, a few lesions appear smaller. Infiltration of immune cells may lead to the appearance of increased tumor size, a phenomenon known as pseudo-progression^9^. Following pembrolizumab monotherapy, there was severe worsening and progression of disease involvement (Fig. 1c). During the dual treatment period, there was substantial regression of melanoma metastases (Fig. 1d).

### DPCP and pembrolizumab have contrasting effects on immune checkpoint protein levels in tissue when used as monotherapy

**Figure 2:**
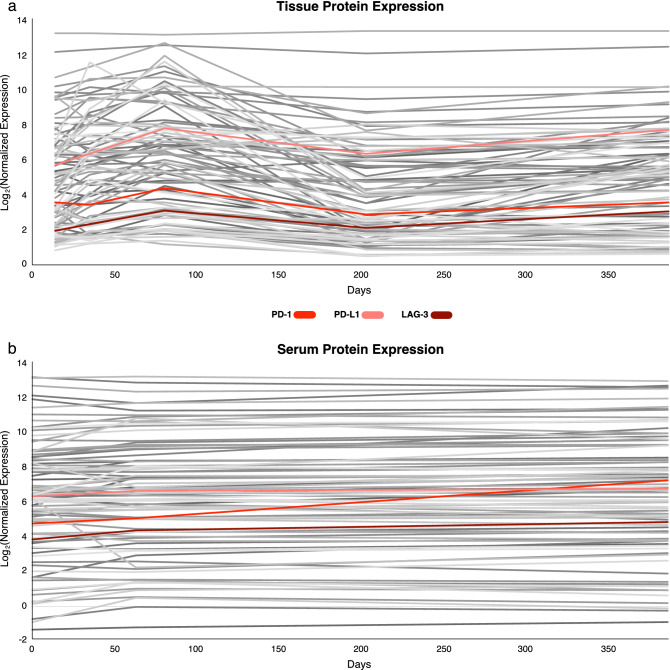
Tissue protein expression (**a**) and serum protein expression (**b**) of all 96 proteins within the Olink immuno-oncology panel shown throughout the treatment course. PD-1, PD-L1, and LAG-3 are highlighted within the set.

Within the tissue samples, protein levels of checkpoint inhibitor proteins PD-1, PD-L1, and lymphocyte-activation gene 3 (LAG-3) rose from baseline until the discontinuation of DPCP monotherapy at Day 80 by 22.7%, 36.9%, and 56.4%, respectively. Pembrolizumab monotherapy was initiated at Day 80. PD-1, PD-L1, and LAG-3 decreased by 19.9%, 11.5%, and 6.7%, respectively, between Day 80 and Day 202 (Figs. 1e–g and 2a).

### Dual therapy with pembrolizumab and DPCP is associated with increased expression of various proteins

**Figure 3:**
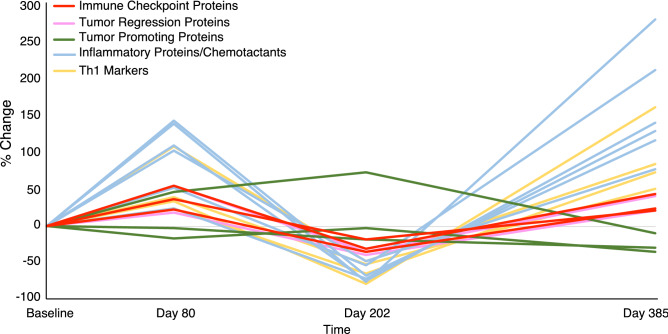
Tissue protein expression of proteins discussed in Table 1, plotted throughout the treatment course. Proteins are color coded by pathway and function. Five groups are represented including immune checkpoint proteins (PD-1, PD-L1, and LAG-3), tumor regression proteins (TNFSF14, GZMA), tumor promoting proteins (CCL17, CD70, LAMP3), inflammatory proteins/chemotactants (CXCL1, IL-6, IL-8, MCP-1, MCP-2, MMP12), and Th1 markers (CXCL9, CXCL10, CXCL11, IFNG).

Dual treatment with pembrolizumab and DPCP was associated with rebound increases of PD-1, PD-L1, and LAG-3 (24.7%, 21.3%, and 44.6%, respectively) between Day 202 and Day 385 (Fig. 1e–g). Serum protein levels for immune checkpoint proteins were not available at the onset of dual therapy, but generally trended upwards during all periods (Figs. 1e–g and 2b). Proteins associated with tumor regression including granzyme A (GZMA) and Th1 markers such as the C-X-C motif chemokine ligand family (CXCL9, CXCL10, CXCL11) and interferon gamma (IFNG) mirrored the checkpoint inhibitor decreases seen in tissue during single agent pembrolizumab therapy. These proteins then rebounded following initiation of dual therapy with DPCP. Tissue protein levels of tumor necrosis factor superfamily member 14 (TNFSF14), a protein with antitumor activity^10^, decreased during pembrolizumab monotherapy by 35.5%, but levels rebounded by 41.2% upon dual therapy initiation. Markers of inflammation and neutrophil chemotaxis^11^ including CXCL1, interleukin (IL)-6, and IL-8 also followed this pattern with a high degree of induction during dual therapy (283.9%, 78.6%, and 215.5%, respectively). Additional markers of inflammation and monocyte chemotaxis including monocyte-chemoattractant protein-1 (MCP-1) and MCP-2 as well as tissue remodeling marker matrix metallopeptidase 12 (MMP12) followed a similar pattern (Table 1 and Fig. 3).

**Table 1:** Proteomic profiling of tissue samples highlighting responses to DPCP monotherapy (baseline to Day 80), pembrolizumab monotherapy (Day 80 to Day 202), and DPCP and pembrolizumab dual therapy (Day 202 to Day 385). Results are depicted as percent change between each time point. Bold text indicates increases in protein level, while italic text indicates decreases in protein level.

Protein	Baseline to Day 80 (DPCP) (% change)	Day 80 to 202 (Pembrolizumab) (% change)	Day 202 to 385 (Dual Therapy) (% change)
LAG-3	**56.4**	*−31.6*	**44.6**
PD-1	**22.7**	*−34.8*	**24.7**
PD-L1	**36.9**	*−18.5*	**21.3**
CXCL1	**141.3**	*−76.2*	**283.9**
CXCL9	**40.8**	*−51.8*	**50.9**
CXCL10	**24.1**	*−64.4*	**74.4**
CXCL11	**34.5**	*−79.2*	**164.2**
GZMA	**18.3**	*−39.6*	**21.6**
IFNG	**109.8**	*−47.7*	**85.7**
IL-6	**104.1**	*−48.5*	**78.6**
IL-8	**53.5**	*−53.7*	**215.5**
MCP-1	**24.18**	*−72.4*	**130.9**
MCP-2	**110.9**	*−74.3*	**143.1**
MMP12	**145.2**	*−67.7*	**118.3**
TNFSF14	**56.4**	*−35.5*	**41.3**
CD70	*−2.91*	*−17.7*	*−30.0*
LAMP3	*−16.5*	*−2.1*	*−35.0*
CCL17	**47.8**	**74.3**	*−10.3*

### Dual therapy with pembrolizumab and DPCP suppresses markers of tumor progression to a higher degree than monotherapy with either agent

C–C Motif Chemokine Ligand-17 (CCL17) is a tumor promoting protein that activates regulatory T cells thereby inhibiting the antitumor response. Levels of CCL17 in tissue samples rose by 47.8% from baseline to Day 80 during DPCP monotherapy and further increased by 74.3% during pembrolizumab monotherapy. In contrast, a 10.3% decrease was observed during dual therapy. Cluster of differentiation-70 (CD70) is a protein implicated in tumor cell survival that also activates regulatory T cells and skews T cells toward exhaustion. Protein levels of CD70 in tissue samples decreased from baseline to Day 80, further from Day 80 to Day 202, and by the highest degree between Day 202 and Day 385 (2.9%, 17.7%, and 30.0%, respectively), as did serum levels from DPCP monotherapy (2.7%) to dual therapy (6.9%). Lysosome associated membrane protein-3 (LAMP3) represents another tumor promoting protein for which the greatest downregulation was observed during dual therapy (35.0%) compared to DPCP monotherapy (16.5%) or pembrolizumab monotherapy (2.1%). Serum levels of LAMP3 mirrored these decreases with downregulation during DPCP monotherapy (7.9%) and further reduction during dual therapy (15.1%) (Table 1 and Fig. 3).

## Discussion

Our findings suggest that, in the context of cutaneous melanoma metastases, DPCP monotherapy is associated with an overall augmentation of immune checkpoint proteins, whereas pembrolizumab monotherapy is associated with a reduction in these proteins. Interestingly, when used in combination, DPCP and pembrolizumab demonstrated a rebound increase in key immune checkpoint markers, and this was clinically associated with regression of cutaneous metastases. The induction of immune checkpoint regulators during dual therapy cannot be attributed to DPCP alone, given the robust clinical improvement during the combined regimen compared to DPCP monotherapy. Considering other successful reports involving PD-1 inhibition (pembrolizumab or nivolumab) and contact sensitization (DPCP or squaric acid dibutylester)^4,5^, our findings provide deeper mechanistic evidence for the potential synergy between agents of these two classes. A proposed mechanism for these findings is that the generation of additional PD-1 substrate by DPCP augments the inhibitory activity of pembrolizumab.

LAG-3 demonstrated the greatest increase among checkpoint proteins during dual treatment. In the context of the recent approval of combined nivolumab and relatlimab (a LAG-3 inhibitor approved for unresectable or metastatic melanoma)^12^ our results demonstrating PD-1 increase and outsized LAG-3 induction from dual treatment may preliminarily indicate a therapeutic role for pembrolizumab and DPCP in multi-agent therapy with LAG-3 inhibitors which merits further investigation.

A greater degree of induction with dual therapy demonstrated in several proteins within our analysis supports the proposed synergistic mechanism. Specifically, markers of immune activation such as the CXCL family and MCP family as well as pro-inflammatory proteins were activated to a higher degree during dual therapy than with monotherapy of either drug. In tandem with inflammatory proteins being upregulated as expected with immune activation caused by DPCP, immune checkpoint proteins were also increased, albeit to a lesser magnitude. This is in line with the known negative feedback role of immune checkpoint proteins during an active immune response^8,13,14^. The augmentation of inflammation with dual therapy may explain, in part, the improved clinical response considering that DPCP functions by inducing a delayed-type hypersensitivity reaction.

Similarly, greater downregulation of tumor promotion proteins such as CCL17, CD70, and LAMP3 during dual therapy suggests that dual therapy further augments tumor suppression. High levels of LAMP3 are implicated in reduced progression-free survival and resistance to chemotherapy and radiation in several cancer types. Therefore, LAMP3 is being studied as a marker of poor prognosis^15^. CD70 functions similarly to immune checkpoint proteins in that it allows tumor cells to evade the anti-tumor immune response. CD70 is being investigated a potential therapeutic cancer target^16^.

The Day 202 time point included one month of dual therapy, indicating a potential limitation to our analysis. However, prior studies on cutaneous melanoma metastases have excluded patients who received one month or less of DPCP treatment^17^. Therefore, data from this time point were considered reflective of single-agent pembrolizumab therapy due to a likely insignificant contribution from DPCP. Additionally, PD-1 serum levels increased over the course of all three treatment phases. It is possible that PD-1 levels decreased during pembrolizumab monotherapy and subsequently increased during dual therapy, but this could not be specifically assessed considering a lack of serum samples during pembrolizumab monotherapy.

While associational, the consistent induction of checkpoint inhibitor and other immuno-oncology proteins and augmented suppression of tumor promoting proteins in the presence of DPCP suggests the potential utility of synergistic immunotherapies in the treatment of cutaneous melanoma metastases. Our analysis is limited to an immuno-oncology panel, as we did not assess proteins that were less likely to be affected by DPCP. Although it is not possible to ascertain significance with samples from a single patient, these observations merit larger-scale investigation and consideration in unresponsive clinical cases.

## Methods

Written, informed consent was obtained from the subject, and ethics approval was granted by The Rockefeller University’s Institutional Review Board (ClinicalTrials.gov number: NCT01711684; 22/10/2012). The study adhered to the Declaration of Helsinki Principles. The subject had no oncologic therapeutic interventions in the 6 months prior to sample acquisition. At the start of the study, the subject applied a topical ultra-pure gel formulation of DPCP evaluated by the FDA to the metastases present on the right leg twice weekly for 80 days. The DPCP was dissolved in an emollient vehicle consisting of isopropyl myristate, surfactant polysorbate 80, polyoxyl stearate, as well as methyl and propyl paraben preservatives. This formulation was selected as it was available as an investigational new drug (IND) and has been used previously in FDA-sanctioned studies. Concentrations alternated between 0.4% and 0.04% to maintain a tolerable level of inflammation. At the discretion of the treating physician, the 0.4% dose could be administered at any office visit if the skin metastases were insufficiently inflamed with the 0.04% concentration. Conversely, doses were held or concentration was lowered if the patient deemed the pruritis, pain, and inflammation associated with the reaction intolerable. Each application of DPCP (occurring twice weekly) was self-administered by the patient such that all cutaneous metastases were covered with a thin layer of gel (the patient was asked to return the tube containing the DPCP gel at each clinic visit for weighing in order to ensure compliance), and then covered with Tegaderm for at least 2 h. At Day 80, DPCP was discontinued and monotherapy with pembrolizumab (2 mg per kg every 3 weeks) was initiated. At day 171, dual therapy with pembrolizumab and DPCP was initiated. Both doses remained consistent with those described.

Tissue samples were taken at Days 14 (baseline before any DPCP was applied), 35, 80 (DPCP monotherapy), 202 (one month of dual treatment), and 385 (seven months of dual treatment) using 6 mm punch biopsies. Samples from Day 202 were considered pembrolizumab monotherapy given prior criteria^17^. The biopsies were bisected, and 10 μg protein from each skin biopsy sample was used for analysis. Serum samples were collected at Day 0, Day 63, and Day 385. Protein expression of both serum and tissue were quantified using the Olink Proseek multiplex ultrasensitive platform immuno-oncology panel (96 biomarkers). Log_2_ of normalized protein expression was plotted over time.

### Ethics approval

This study was approved by The Rockefeller University’s Institutional Review Board, approval #JKR-0788, ClinicalTrials.gov listing: NCT01711684. Written, informed consent was obtained, and the study adhered to the Declaration of Helsinki Principles.

### Informed consent

Informed consent was received from the subject for publication of identifying information/images in an online open-access publication.

## Data Availability

The datasets used and/or analyzed during the current study are available from the corresponding author, Nicholas Gulati, on reasonable request.
